# An analytical assessment of industrial sector innovative management in the context of digitalization

**DOI:** 10.1186/s13731-022-00247-y

**Published:** 2022-10-14

**Authors:** Nurzhanat Sherimova, Baurzhan Isabekov, Miras Alkeev, Zhanna Yermekova, Tatyana Ostryanina

**Affiliations:** 1 Department of Management, L.N. Gumilyov Eurasian National University, Nur-Sultan, Kazakhstan; 2 Department of Geography and Tourism, S. Toraighyrov Pavlodar State University, Pavlodar, Kazakhstan; 3 Department of Finance and Accounting, Al-Farabi Kazakh National University, Almaty, Kazakhstan; 4 Department of Economics, Kostanay Engineering and Economics University Named After M. Dulatov, Kostanay, Kazakhstan

**Keywords:** Management transformation, Industry, Technology, Innovation management, Digitalization

## Abstract

The accumulated potential in digitalization suggests the need to create a new paradigm for managing scientific-innovative and production-technological processes, which is reflected in the author’s article. In these conditions, there is the problem of developing a mechanism for innovative management of the industrial sector of the economy. The study aims to analyze statistical and analytical data of modern industrial sector management in the context of digitalization. By analytical, comparative, and statistical analysis of international innovation management approaches, according to the rating of the global innovation index 2020/2021 and business activity of technological leaders in Asia, North America, and Europe, the authors developed a methodological approach to improve the mechanism for implementing innovative management in industrial sector. The mechanism includes such core elements: state industrial policy—purposes of industrial development—decision on innovative management implementation—development of mechanism to implement innovative decision—expected short- and long-term results based on the traceability of innovation and the overall economic context from a global perspective. The study results can be applied for implementing innovative management in industrial sector and developing industrial policies.

## Introduction

In the modern world, industry is designed to meet the needs of the life of the entire population of the state, as well as the realization of its internal and external interests. The fundamentally new conditions prevailing in the twenty-first century dictate the implementation of scientific and technological modernization of economic sectors, while responding to all the challenges of competition. The industrial sector, its activities and development, are significant in this process. This sector covers material, human resources, industry management bodies and infrastructure. All this in aggregate is a mechanism requiring timely tactical and strategic management policies.

The transition to a post-industrial society that accelerated the implementation of scientific and technical progress, strengthening the processes of uncertainty and risks, crisis situations, requires a constant search for new management solutions. The innovativeness of management decisions is an objective necessity in modern conditions. In this regard, the success of industrial enterprises depends on the formation of a fundamentally new innovative approach to solving problems, which in general allows achieving positive effects and results in an innovative economy.

The universally recognized world concept for the development of the industrial sector is recognized as “digitalization”. The initiative, proclaimed by the German luminaries, later taken up by the United States and Southeast Asia, has given significant shifts in the introduction of end-to-end digitalization, fundamentally new high-tech installations and programs.

## Literature review

The 2019–2020 coronavirus pandemic and the associated global economic collapse have demonstrated that decision-makers around the world are ill-equipped to identify the innovative possibilities of modern societies (Bowden et al., 2020; Rousseau, 2018). Researchers argue that the right solution to the problem of management lies in the study of innovation management development, which is understood as the systematic promotion of innovation in the organization, affecting the result and the receipt of benefits (Drucker, 2020; Hengsberger, 2018). Management structures can be divided according to the signs of centralization: centralized and decentralized, according to the functional orientation: sectoral, functional, programmed (Cavatorto & Spina, 2020). An analysis of management in highly developed countries (Norris, 2016; Oqubay et al., 2020; Pianta et al., 2020) found that a functional industrial management system has a special effect in modern conditions, in which the goal of innovation is to create additional value within the organization. The experience of national economies of key centers of the world, which include the USA, Japan, the EU, and China, has shown that active changes in industrial policy towards scientific and technological shifts, the transition to modernization, and increased investment in basic and applied research have largely predetermined their current advanced state of the economy (Norris, 2016; Oqubay et al., 2020; Pianta et al., 2020). This concept is consistent with the approach of Andreoni, 2016; Di Tommaso et al., 2020 to the study of industrial policy in developed countries, which aims to create and develop sectors of the economy identified as priority. Aggarwal & Reddie, 2020 take a similarly broad view of macro-level innovation governance, taking into account the economic aspects of strategic competition, arguing that innovation governance, transformed in the current era, is becoming a central aspect of geostrategic considerations. As part of their work, the researchers identified four trends in the industrial sector: a reduction in the number of people employed in industrial production, an increase in the automation of production, the demand for a highly skilled workforce, the growth of the share of high-tech industries. Liu & Liu, 2019 also studied the experience of advanced countries, which showed that intensive technological development is impossible without the use of modern management methods and organization of production. More precisely, the mutual combination of new technologies and new management mechanisms has allowed countries to increase indicators of industrial development and accelerate positive structural changes associated with the growth of high-tech and knowledge-intensive industries (Liu & Liu, 2019). Moreover, analysis of developed countries’ experience in industry digital transformation showed that among the main concepts there are: Smart Production, Digital Production, Industry 4.0, Internet in Industry. They stimulate effective management decisions at all levels, from the state to the private, from the macro- to the micro-levels (Bueno et al., 2020). This thesis is confirmed by Gaidarova, 2016. The researcher denotes that in modern market conditions the innovative orientation of industrial enterprises increases the consumption of products and contributes to the balance and efficiency of the markets as a whole. Gaidarova proposes to evaluate the result of innovation management by a system of indicators that characterize the effectiveness of business decision-making practices, such as the ability to innovate, the quality of this work implementation, innovation activity of the enterprise, product competitiveness. The researcher believes that with this approach, innovation can be considered the key to sustainable economic development. Another group of researchers (Bowden, 2020; Cummings et al., 2017) considered innovation management through three models in management, European, American, and eastern. The European model (Germany as an option) focuses on the standardization of all processes, on the integration of European communities digitally. The Anglo-American or Western model (USA, Canada and Great Britain) is associated with the exclusion of excessive state functions and the high efficiency of decisions. The Eastern, Asian model is based on a multi-level hierarchy of the management system. This model pays great attention to the individual, education, culture, reduction of the state apparatus. As practice shows, the USA and developed EU countries: Great Britain, France, Germany, and the Netherlands are recognized leaders in innovation development in the modern world. In this regard, in terms of searching for a vector for the development of innovative management, the “Western sector” seems to be traditional, rich in terms of the proposed tools and directions of development (Dutta et al., 2018). It should be noted the positive experience of Asian countries to integrate into a global network of innovation and entrepreneurship, based on the increase in the consumer market and information sector (Bhagavatula et al., 2019; Das et al., 2020; Shukla, 2017). Particular success was achieved in this direction by South Africa—a country with rich natural and labor resources, which focused industrial policy emphasis on innovation in disadvantaged rural areas to accelerate local economic development and public services (Booyens & Hart, 2019). It is difficult to quantify precisely the “external effect” from the application of new management technologies, but their role in the qualitative transformation is undeniable. All countries in different ways have been able to adapt the new management methods. However, the priority in the field of applying innovative solutions is the transition from quantity to quality of innovation (Dutta et al., 2019), which is aimed at obtaining high economic, social, and environmental results (Tsindeliani et al., 2021).. Scientific research in the field of studying the concepts, processes and mechanisms of innovative management, digitalization and industrial policy was created and developed in the works of numerous scientists and practitioners. They justified relevance, developed a mechanism, ways to improve the manageability of these processes. Despite the existing works on the topic, there is no analytical assessment of innovation management in the industrial sector, which would reflect the extent of innovation activity in the industry and could be used in various analytical comparisons, especially in developing countries, where the role of innovation impact in the management and tactics of production development is increasing. The significance of this study is to consider innovation management in the overall context of the economy from a global perspective.

Practical contribution is the development of a universal mechanism of industrial innovation management based on the use of benchmarking tool, which allows one to improve the practice of business decisions at the meso- and macro-level in the global changes in science and technology.

The aim of the study: analysis of statistical and analytical data of modern practices of industrial sector management in the context of digitalization.

For the purposes of the study the following tasks were performed:

1. Identification of main principles of innovative management in industrial sector.
2. Investigation of experience and identification of principal guidelines and peculiarities in innovative industrial policies of world economies.
3. Development of a mechanism for implementing innovative management in industrial sector.

## Material and methods

The study builds on previous research on approaches to innovation management in the context of digitalization and sectoral policies of the world's economies, drawing on changes in the global innovation index (GII), which helps to make innovation meaningful for countries, especially developing countries and assess the relative effectiveness of the national innovation system in the innovation activities of industrial enterprises. The theoretical part of the study is based on the concepts of: Bowden et al. (2020), Bueno et al., (2020), Sherimova, (2019). In its empirical part, the study used the reports of the international organization WIPO, 2020/2021 and WIPO Statistics Database, 2022.

The study was conducted in three stages:

*Stage 1*. The authors constructed a block diagram of innovation management using the Data Table function of Excel program and Microsoft Visio graphic editor to visualize the basic innovation management principles in industrial enterprises, which are linked to the literature (Bowden et al., 2020; Bueno et al, 2020; Sherimova, 2019). The scheme of innovation management includes Block 1: the subject of management and Block 2: the object of management, which in the process of management transformation lead to the synergy of results.

*Stage 2*. The study systematized the relationship of managerial approaches in industrial business practices and digital technologies based on evaluating GII in the context of four quartiles (I quartile—performance above the expected level of economic development; II—performance in accordance with the level of economic development; III and IV—all other economies). The overall GII score is the average of the input (innovation input) and output (innovation output) sub-indices, which are used to rank 132 economies representing 94.3% of the world’s population and 99.0% of global GDP at purchasing power parity in current international dollars. The result of this step is presented as a summary table in Excel, which is created from the report data (WPO, 2020/2021). For each economy, the most recent annual data available in the national statistics of the analyzed countries are taken into account. The result processing formula for the GII model is as follows: $\mathrm{In}\left[\frac{\left(\mathrm{max}\,xf-1\right)\times \left(\mathrm{max}-economy \,value\right)}{\mathrm{max}-\mathrm{min}+1}\right]$, where “min” and “max”—minimum and maximum values of the indicator sample.

*Stage 3*. Based on assessing the innovation potential of business practices in the context of GII (WPO, 2020/2021) and activity of the world innovation process leaders in the issues under consideration according to the Hague system of international registration of industrial designs (WIPO Statistics Database, 2022), which covers 93 countries, a universal mechanism of innovation management implementation in the industrial sector was developed. This mechanism emphasizes the achievement of business and socio-economic value as measured by traditional criteria and the overall economic context of the GII function, the global innovation tracker, including the amount of investment in science and innovation, technological process, and socio-economic impact.

The methodological basis of this study is represented by analytical, comparative, and statistical analysis.

## Results

In a complex and rapidly changing external environment, national innovation systems began to pay attention to the problems of changing the practice of innovation management to develop the industrial sector. Literature review showed that the search for ways of innovative management as part of the state economy is based on the two main blocks of management transformation:

- Block I—improving the organizational and managerial apparatus;
- Block II—the introduction of modern management technologies.

**Fig. 1:**
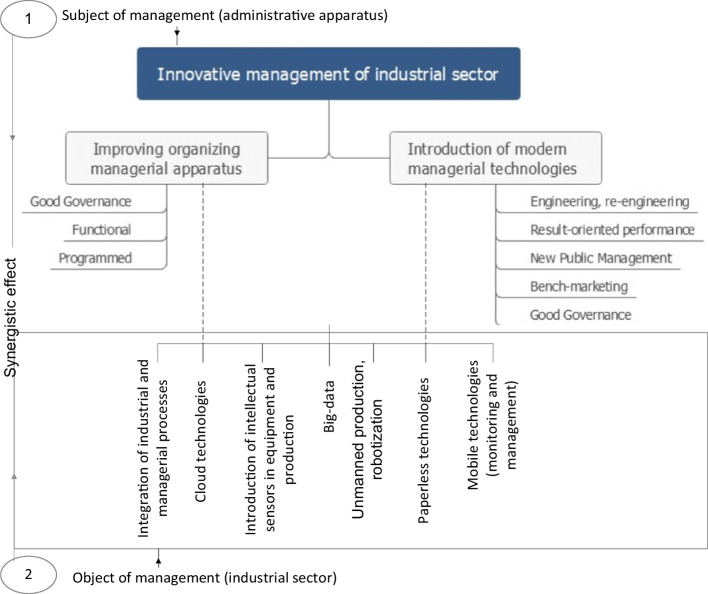
Innovative management of industrial sector. *Source:* developed by the authors based on Sherimova (2019), Bowden et al. (2020), Bueno et al. (2020)

Figure 1 shows industrial sector innovative management and in general includes the principle of systemicity—the totality of all management process elements, which are in constant development and interaction; digitalization—the process of transforming information using digital technologies, the implementation of which affects the scale of industrial development; business processes transformation based on the distribution of new knowledge and technologies aimed at meeting the needs of society and the state.

The essence of the mechanism of innovative management in the industrial sector (Fig. 1) is that in the course of management, the subject (managerial staff—all levels of management) affects in various ways (new management methods) the management object (industrial sector) for synergistic results, which entails the joint achievement of business and socio-economic values. Since the development of the methodology for managing the industrial sector, in the context of disseminating new knowledge and technology, is a very urgent task, it is of interest to measure the innovative potential of business practices and economic development, which ensures the effectiveness of decision-making in government, business, and other areas, as it seeks to develop policies to produce effective approaches and mechanisms for building innovative management using international experience. It is also important to determine the extent to which they can be used to improve decision-making procedures for the formation and adjustment of industrial policy in the current environment for many developing countries.

Table 1 presents the experience of international practices in the field of innovation as of 2021.

**Table 1:** The innovative potential of business practice in the general context of the economy: 2020—2021

Best/average/last rank 2021	4th quartile (ranks 1st to 33rd)	Score (0–100)	Changes to rank 2020	Best/average/last rank 2020	3rd quartile (ranks 34th to 66th)	Score (0–100)	Changes to rank 2020
1	Switzerland	65.5	0	34	Hungary	42.7	+ 1
2	Sweden	63.1	0	35	Bulgaria	42.4	+ 2
3	United States of America	61.3	0	36	Malaysia	41.9	− 3
15	Hong Kong, China	53.7	− 4	49	Ukraine	35.6	− 4
16	Israel	53.4	− 3	50	Montenegro	35.4	− 1
17	Canada	53.1	0	51	Philippines	35.3	− 1
31	Portugal	44.2	0	64	Republic of Moldova	32.3	− 5
32	Slovenia	44.1	0	65	Uruguay	32.2	+ 4
33	United Arab Emirates	43.0	+ 1	66	Saudi Arabia	31.8	0

Best/average/last rank 2021	2nd quartile (ranks 67th to 99th)	Score (0–100)	Changes to rank 2020	Best/average/last rank 2020	1st quartile (ranks 100th to 132nd)	Score (0–100)	Changes to rank 2020
67	Colombia	31.7	+ 1	100	Namibia	24.3	+ 4
68	Qatar	31.5	− 2	101	Guatemala	24.1	+ 5
69	Armenia	31.4	− 8	102	Rwanda	23.9	− 11
79	Kazakhstan	28.6	− 2	114	Côte d’Ivoire	21.0	− 2
80	Azerbaijan	28.4	+ 2	115	Burkina Faso	20.5	+ 3
81	Jordan	28.3	0	116	Bangladesh	20.2	0
97	Trinidad and Tobago	24.8	+ 1	130	Guinea	16.7	0
98	Kyrgyzstan	24.5	− 4	131	Yemen	15.4	0
99	Pakistan	24.4	+ 8	132	Angola	15.0	No data

*Source:* Dutta et al. (2020, 2021)

**Fig. 2:**
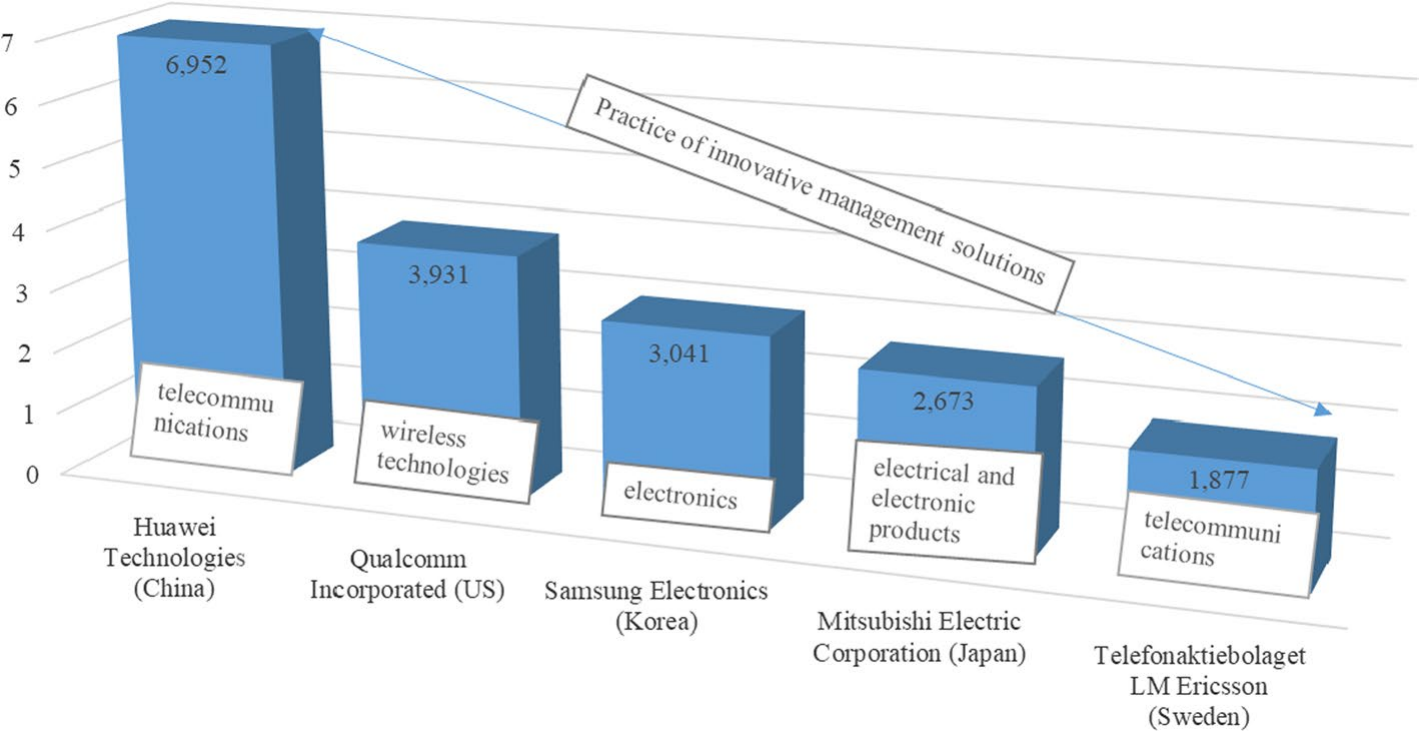
Leaders of the global innovation process in the industrial sector, 2021. *Source:* WIPO Statistics Database (2022)

Analysis of Table 1 shows that the global innovation landscape in 4th and 3rd quartile groups is changing slowly compared to the 2nd and 1st quartiles of the GII ranking for the period 2020–2021. This shows the priority of innovation in the socio-economic development of these countries, especially in North America and Europe, which continue to lead and have the strongest and most balanced innovation systems. Moreover, one should note the developing innovation sphere in Asia, as evidenced by the places that countries such as China, Israel, the UAE, and Malaysia occupy in the GII. The most important need for the development and implementation of innovative industrial policy is to stimulate innovation activity at the meso- and macro-level. This aspect of practice contributes to the formation of qualitatively new competitive positions, providing technological priority of business activity, which is confirmed by the results achieved by the world leaders of the industry, according to the Hague system of international registration of industrial designs (Fig. 2):

As shown in Fig. 2, in 2021 the leader in international filings in the industrial sector is Chinese telecommunications equipment manufacturer Huawei Technologies, which accounts for 6952 or 21.2% of all filings in global activity. It is followed by Qualcomm Incorporated (US), Samsung Electronics (Korea), Mitsubishi Electric Corporation (Japan), Telefonaktiebolaget LM Ericsson (Sweden). Thus, one can state that the practice of innovative management solutions at the macro-level effectively influences the industry process of China, the U.S., Sweden to enable consumers and stimulate digital transformation to create an intelligent society.

**Fig. 3:**
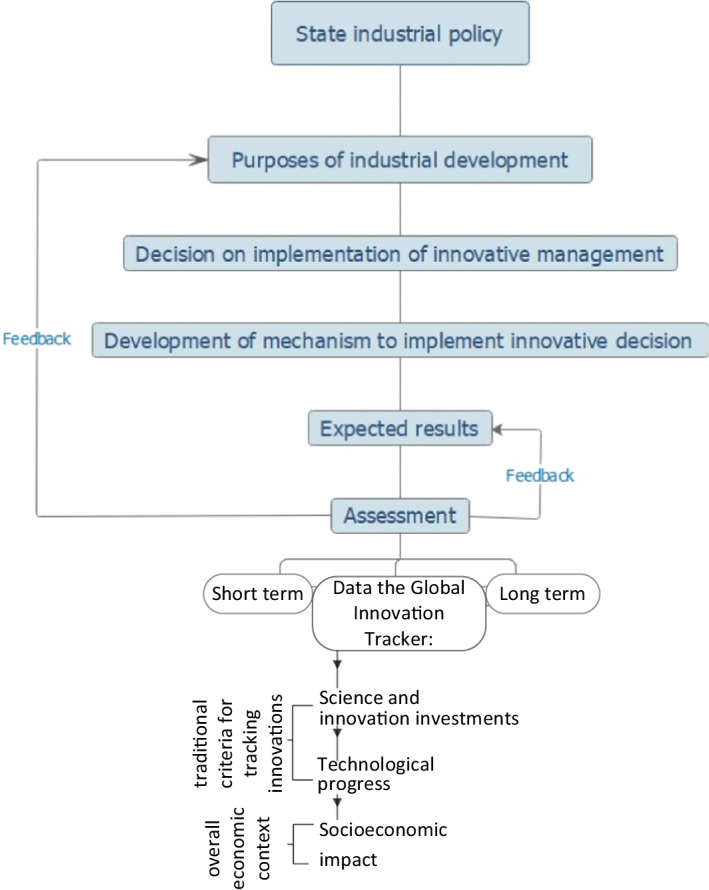
Mechanism for implementing innovative management in industrial sector. *Source:* developed by the authors based on Dutta et al. (2020), Dutta et al. (2021), WIPO Statistics Database (2022)

In accordance with the analysis results, the mechanism of managerial decisions’ effectiveness in terms of achieving industrial policy strategic objectives was developed, which involves such elements as input, output, and evaluation (short-term and long-term). In particular, the estimated results are built on the indicators of the GII function (Global Innovation Tracker), which reflects key trends in innovation management through three stages of the innovation process: the amount of investment in science and innovation; technological progress; socio-economic impact (Fig. 3).

As shown in Fig. 3, the effect of implemented management decisions is evaluated on the basis of traditional criteria—the amount of investment in science and innovation (international patent applications), technological progress (renewable energy sources), as well as the overall economic context—socio-economic impact (labor productivity). Consequently, the estimated results as a management mechanism contain basic data in the field of innovation, which makes the mechanism in practice universal.

In general, the conducted analytical assessment regarding the practice of development and implementation of new management aspects in industrial policy based on the experience of leading Western countries allows one to highlight a number of key strategic directions of innovative transformations:

- firstly, comprehensive scientific support of sectoral transformations due to transformation of management in the context of interacting management structures to achieve synergy of results;
- secondly, creating a system of motivation for the growth of business activity in the industrial sector based on international practices in the field of innovation and leaders of the global innovation process;
- thirdly, implementation of innovation management in accordance with the industry priorities of the national economy from a global point of view.

## Discussion

One of the study results is an analytical assessment of current practices of industrial innovation management. Of central importance is the collection of statistical and analytical data, choosing and deciding which ideas will be implemented to develop a mechanism for implementing innovation management in this sector. This process is built on the experience of international practice in the field of innovation and the activity of the leaders of the global innovation process. It is presentation of information that combines all the signs of classification that most accurately represent and characterize the description of managerial experience, their phased presentation and a brief summary of the main directions of managerial paradigms, which is also confirmed in the work of Bowden, 2020. After analyzing the temporal and substantive foundations of managerial aspects, the researcher concluded that the gradual transition from the old trajectory to the new more democratic ones, as a result, brought many states to the effectiveness and efficiency of management technologies. With regard to changes at the industry and inter-industry level, the following should be noted here: the digital transformation of the industry leads to the formation of a customer–consumer relationship model through technologies aimed at customer needs. Priority to the customer allows you to combine the capabilities of physical, digital and human resources through the optimization of production. The use of new digital technologies in industry requires a corresponding change in corporate and organizational structure, as well as professional skills. These elements should change towards a flexible hierarchy of new parameters, measuring results, as well as new strategies in management (Bueno et al., 2020). Researchers (Aiginger & Rodrik, 2020; Khin & Ho, 2019) note that in the context of digitalization all the leading countries of the world are forming their own strategies in the field of the digital economy, are embarking on the path of digitalization. Today, 80% of the participating countries (total 34 countries, 2015) of the Economic development and cooperation in the digital economy program have formed their national strategy and course for the development of the digital economy. Several participating countries do not yet have a common strategy, but have already begun work in this direction. Among all countries and regions, the strategies of the USA, EU and Japan are significant. Based on the experience of developed countries, it was revealed that basically these countries relied on the presence of two main blocks—consideration of the organizational structure of management and the use of new methods and technologies, which led to positive trends in general economic performance, which is consistent with the results of Petry, 2018. Similar to the present study, Andreoni, 2016 analyzed and compared different industrial policies, focusing on the large advanced economies of the United States, Japan, and Germany, whose industrial policies have historically represented "learning benchmarks" in their respective continental regions, and found that industrial policy measures contribute to the growth of industrial production. Aggarwal & Reddie, 2020 indicate that industrial policy with a focus on digitalization in management is a new form of economic and social development, which has replaced the outdated realities. The new management paradigm is based on the tactics of conducting the national economy, where knowledge and information are digitized, and as a key production factor is the driving force of productivity and optimization of the structure of the economy (Aggarwal & Reddie, 2020). In particular, the industrial sector is evolving with the development of digital management technologies. Its content and direction are changing; the result of the new management should be a change in the classification of sectors of the national economy. It should be noted that the main industries on which the digital revolution is based are the production of computer and communication equipment, electronic equipment, telecommunications, software, information technology services, etc. Almost all industries built on digital technology can be considered the scope of the digital industry. Today, the synthesis of questions about the digital industries and the new management techniques used in them has a wide discussion (Li, 2020). One of the results of the study revealed that the digitalization of public administration at all levels is the basis for the growth of its effectiveness. Moreover, the management mechanism should be designed in such a way that the elements involved in it, from the initial data to the exit from the entire process, are aimed at implementing and achieving the main goal of the industrial sector (Tassinari et al., 2019). It is quite natural that to build a general scheme of the mechanism for implementing innovative management in the industrial sector, the experience of international practices in the field of innovation and the business activity of the global innovation process leaders were taken into consideration. The common characteristics of strategic innovation transformations in the industry of these countries are the scientific support of industrial transformations, the improvement of organizational infrastructures, the structural restructuring of the entire industry, the creation of a solid investment base, and the revision of the personnel system in accordance with the priorities set (Andreoni, 2016; Pianta et al., 2020). Nevertheless, there are possible risks at the stages of its application and implementation. It is necessary to find such mechanisms that could lead to significant shifts in scientific and innovative development with an emphasis on priority areas. However, there may be “undercurrents”. Relying on focusing on priority development trajectories, it is necessary to take into account real needs, specific projects in sectors, and achievement of socially vulnerable goals (Di Tommaso et al., 2020). The solution to these problems is closely related to the development and maintenance of innovatively active enterprises with a solid institutional base. In this study, to solve management problems in the context of digitalization, the authors studied the activity of world technology leaders Huawei Technologies (China), Qualcomm Incorporated (US), Samsung Electronics (Korea), Telefonaktiebolaget LM Ericsson (Sweden), which is based on creative approaches, finding new solutions, activating scientific factors towards full digitalization, which agrees with the results of Heavin and Power (2018); Gerrikagoitia et al., (2019). Thus, one can state that new trends in management transform traditional management processes and make the transition from a centralized to a decentralized plane (Tsindeliani et al., 2021b). This completely changes the entire management process on the path of digital transformation. In this framework, the authors have developed a modern methodological management approach, which is based on three innovation process stages in the context of tracking global trends in innovation based on the GII rating and its function—global innovation tracker—with an emphasis on applying a benchmarking tool.

## Conclusion

The study accomplished the tasks and achieved the aim. By way of analytical, comparative, and statistical analysis of management approaches in context of digitalization and innovations, based on international experience of the world's economies according to the GII rankings 2020/2021 and the activity of the global innovation process leaders in Asia, North America, and Europe, the study developed universal mechanism for implementing innovative management in industrial sector. The mechanism includes such core elements: state industrial policy—purposes of industrial development—decision on implementation of innovative management—development of mechanism to implement innovative decision—expected results—assessment (short-term and long-term) based on the data of the GII function—the global innovation tracker.

The results of the research can serve the development of the paradigm of industrial sector management in the digital economy, the basis of which is a universal mechanism focused on meeting the socio-economic needs of society and the state. The management mechanism is defined by the author as the unity of subsystems that are closely interconnected, ensuring viability and mutual development. Subsystems include the interaction of subjects and objects of management, which entails the synergy of the result, which contributes to the creation of business and socio-economic value. Subsystems include the formation of subjects of the digital economy, based on economic and partnership relations for the design, development and use of technology objects, networks for the transmission, receipt and storage of information, digitalization tools, the development, maintenance, improvement and use of which brings added value.

The results of the study can be applied by top-managers and officials for implementing innovative management in industrial sector and developing industrial policies at various levels.

## Data Availability

Data will be available on request.

## References

[CR1] Aggarwal VK, Reddie AW (2020). New economic statecraft: Industrial policy in an era of strategic competition. Issues & Studies.

[CR2] Aiginger K, Rodrik D (2020). Rebirth of industrial policy and an agenda for the twenty-first century. Journal of Industry, Competition and Trade.

[CR3] Andreoni A, Stiglitz JE, Noman A (2016). Varieties of industrial policy. Efficiency, finance and varieties of industrial policy.

[CR4] Bhagavatula S, Mudambi R, Murmann JP (2019). Innovation and entrepreneurship in India: An overview. Management and Organization Review.

[CR5] Bofinger P (2019). Industrial policy: Is there a paradigm shift in Germany and What does this imply for Europe. Social Europe.

[CR6] Booyens I, Hart TGB, Knight J, Rogerson CM (2019). Innovation in a changing South Africa: Extant debates and critical reflections. The geography of South Africa.

[CR7] Bowden B, Bowden B, Muldoon J, Gould AM, McMurray AJ (2020). Management history in the modern world: An overview. The Palgrave handbook of management history.

[CR8] Bueno A, Filho MG, Frank AG (2020). Smart production planning and control in the Industry 4.0 Context: A systematic literature review. Computers & Industrial Engineering.

[CR9] Cavatorto S, La Spina A, Cavatorto S, La Spina A (2020). Introduction: The puzzle of administrative change. The politics of public administration reform in Italy.

[CR10] Cummings S, Bridgman T, Hassard J, Rowlinson M (2017). A new history of management.

[CR11] Das A, Dash DP, Sethi N (2020). Innovation, corruption, and economic growth in emerging Asia. Buletin Ekonomi Moneter Dan Perbankan.

[CR12] Di Tommaso MR, Tassinari M, Ferrannini A, Pressman S (2020). Industrial policy and societal goals. A new look at the American case (from Hamilton to Obama and Trump), Chapter 8.

[CR13] Drucker PF (2020). The essential Drucker.

[CR14] Dutta, S., Lanvin, B., León, L., Wunsch-Vincent, S. (2021). Global Innovation Index 2021. Tracking Innovation through the COVID-19 Crisis, 14th Edition, WIPO, 226 p.

[CR15] Dutta, S., Lanvin, B., Wunsch-Vincent, S. (2020). Global Innovation Index 2020. Who Will Finance Innovation? 13th Edition, WIPO, 448 p.

[CR16] Gaidarova, V. (2016). Innovations in industrial enterprises. International Scientific Journal “Symbol of Science”, No. 4, pp. 51–55. ISSN 2410-700X. https://cyberleninka.ru/article/n/innovatsii-na-promyshlennyh-predpriyatiyah

[CR17] Gerrikagoitia JK, Unamuno G, Urkia E, Serna A (2019). Digital manufacturing platforms in the Industry 4.0 from private and public perspectives. Applied Sciences.

[CR18] Heavin C, Power DJ (2018). Challenges for digital transformation–towards a conceptual decision support guide for managers. Journal of Decision Systems.

[CR19] Hengsberger, A. (2018). Definition Innovation Management. Innovation goal. https://www.lead-innovation.com/english-blog/definition-innovation-management

[CR20] Kenderdine T (2017). China's industrial policy, strategic emerging industries and space law. Asia & the Pacific Policy Studies.

[CR21] Khin S, Ho TCF (2019). Digital technology, digital capability and organizational performance. International Journal of Innovation Science.

[CR22] Li F (2020). Leading digital transformation: Three emerging approaches for managing the transition. International Journal of Operations & Production Management.

[CR23] Liu F, Liu R (2019). China, the United States, and order transition in East Asia: An economy-security Nexus approach. The Pacific Review.

[CR24] Mustar P (2016). Industrial policy in France: In search of lost time. Economia e Politica Industriale.

[CR25] Norris WJ (2016). Chinese economic statecraft: Commercial actors, grand strategy, and state control.

[CR26] Oqubay A, Cramer C, Chang H-J, Kozul-Wright R (2020). The Oxford handbook of industrial policy.

[CR27] PCT top 10 applicants. Which firms lead PCT International Patent Filings. Facts and Figures, WIPO Statistics Database, 2022. https://www.wipo.int/edocs/infogdocs/en/ipfactsandfigures/

[CR28] Petry T, North K, Maier R, Haas O (2018). Digital leadership. Knowledge management in digital change.

[CR29] Pianta M, Lucchese M, Nascia L (2020). The policy space for a novel industrial policy in Europe. Industrial and Corporate Change.

[CR30] Rousseau J-J (2018). Rousseau: The social contract and other later political writings.

[CR31] Schrock G, Wolf-Powers L (2019). Opportunities and risks of localised industrial policy: The case of ‘maker-entrepreneurial ecosystems’ in the USA. Cambridge Journal of Regions, Economy and Society.

[CR32] Sherimova, N. (2019). Innovative management of Kazakh industrial sector in the conditions of digitalization: relevance and content. Bulletin of Karaganda University, series “Economics” № 1 (93), 150-159. https://rep.ksu.kz/bitstream/handle/data/7490/Sherimova_Innovacionnoe_150-159.pdf?sequence=1&isAllowed=y

[CR33] Shukla S (2017). Innovation and economic growth: A case of India. Humanities & Social Sciences Reviews.

[CR34] Tassinari M, Barbieri E, Morleo G, Di Tommaso MR (2019). Targeted Industrial policy and government failures: Insights from the South Korean experience. International Journal of Emerging Markets.

[CR35] Tsindeliani, I., Egorova, M., Vasilyeva, E., Bit-Shabo, I., & Kikavets, V. (2021a). Collection of Taxes from Ultimate Beneficiaries: Russian Regulatory Model. *Accounting, Economics, and Law: A Convivium.*

[CR36] Tsindeliani IA, Lyutova O, Anisina K, Migacheva E, Lesina L (2021). Current trends in counteracting thin (insufficient) capitalization in the Russian legal system. Intertax.

[CR37] Wilks S, Wright M (2016). The promotion and regulation of industry in Japan.

